# Synchronous Melanoma and Follicular Lymphoma in the Same Nodal Basin: A Diagnostic and Therapeutic Challenge

**DOI:** 10.1002/cnr2.70363

**Published:** 2025-10-13

**Authors:** Silvia Borriello, Umberto Santaniello, Matteo G. Brizio, Paolo Fava, Giovanni Cavaliere, Giulia Carpentieri, Rebecca Senetta, Adriana Lesca, Simone Ribero, Pietro Quaglino, Franco Picciotto

**Affiliations:** ^1^ Section of Dermatology and Venereology, Department of Medical Sciences University of Turin Turin Italy; ^2^ Section of Dermatology and Venereology, Department of Medical Sciences AOU Città della Salute e della Scienza Turin Italy; ^3^ Pathology Section, Department of Oncology AOU Città della Salute e della Scienza Turin Italy; ^4^ Division of Nuclear Medicine, Department of Medical Science University of Turin Turin Italy; ^5^ Dermatologic Surgery Section, Department of Surgery AOU Città della Salute e della Scienza Turin Italy

**Keywords:** follicular lymphoma, melanoma, synchronous neoplasm

## Abstract

**Background:**

The incidence of both malignant melanoma (MM) and non‐Hodgkin lymphoma (NHL) has risen in recent decades, with studies suggesting a potential bidirectional association. Nonetheless, synchronous presentation of active disease in both entities remains rare.

**Case:**

We report the case of a 62‐year‐old man with a history of indolent B‐cell lymphoma, exhibiting features between marginal zone and follicular subtype, previously treated with R‐CHOP, radiotherapy, and Rituximab maintenance, achieving complete remission. Ten years later, he developed an ulcerated superficial spreading melanoma (Breslow thickness 4.5 mm, pT4b), with staging CT revealing right axillary lymphadenopathy. Biopsy confirmed relapsed follicular lymphoma. Surgical management included wide excision and sentinel lymph node biopsy, which identified melanoma metastasis in one sentinel node and confirmed FL in the non‐sentinel node. Molecular testing showed a BRAFV600E mutation. The patient received axillary radiotherapy followed by adjuvant BRAF/MEK inhibitor therapy.

**Conclusion:**

At 6‐month follow‐up, imaging showed no evidence of relapse. This represents the first documented case of synchronous stage III BRAF‐mutated MM and FL managed with targeted BRAF/MEK inhibitors combined with localized radiotherapy. The successful outcome validates this sequential multidisciplinary approach and underscores the importance of dermatologic surveillance in NHL survivors.

## 1 Introduction

The incidence of both malignant melanoma (MM) and non‐Hodgkin lymphoma (NHL) has shown a notable rise over the past two decades. Beyond their increasing prevalence, an interesting body of epidemiological evidence has established a bidirectional association between these two malignancies. Large population‐based cohort studies have quantified this link, demonstrating a statistically significant increased risk of developing NHL among MM survivors, with a standardized incidence ratio (SIR) of 2.01, and a similarly elevated risk of MM among NHL survivors, with an SIR of 1.41 [1, 2]. Several hypotheses have been proposed to explain this association. One leading theory centers on a common foundation of immune dysregulation. Chronic immunosuppression, whether disease‐related (as seen in lymphoproliferative disorders) or iatrogenic, is a well‐established risk factor for both MM and NHL [3]. Treatments for lymphoma, such as cytotoxic chemotherapy or anti‐CD20 agents like rituximab, can impair immune surveillance, potentially creating a permissive environment for the development of a second primary malignancy like melanoma [3]. Other potential mechanisms include shared genetic predispositions or common environmental risk factors, although the role of ultraviolet (UV) radiation in NHL pathogenesis remains debated [3, 4]. Despite the recognized epidemiological link, the synchronous presentation of active MM and NHL in the same patient is rarely described in the literature [5]. The concurrent diagnosis of two distinct malignancies within the same neoplastic lesion—a phenomenon known as a collision tumor—is an even more exceptional event [6], with just four cases of collision between MM and NHL reported by Parra‐Medina et al. [7]. Such cases present profound diagnostic and therapeutic challenges, often confounding initial staging and complicating treatment planning. Herein, we report a rare case of synchronous metastatic melanoma and follicular lymphoma (FL) discovered within the same axillary nodal basin, highlighting the critical clinical and pathological considerations in managing such complex presentations.

## 2 Case

**FIGURE 1:**
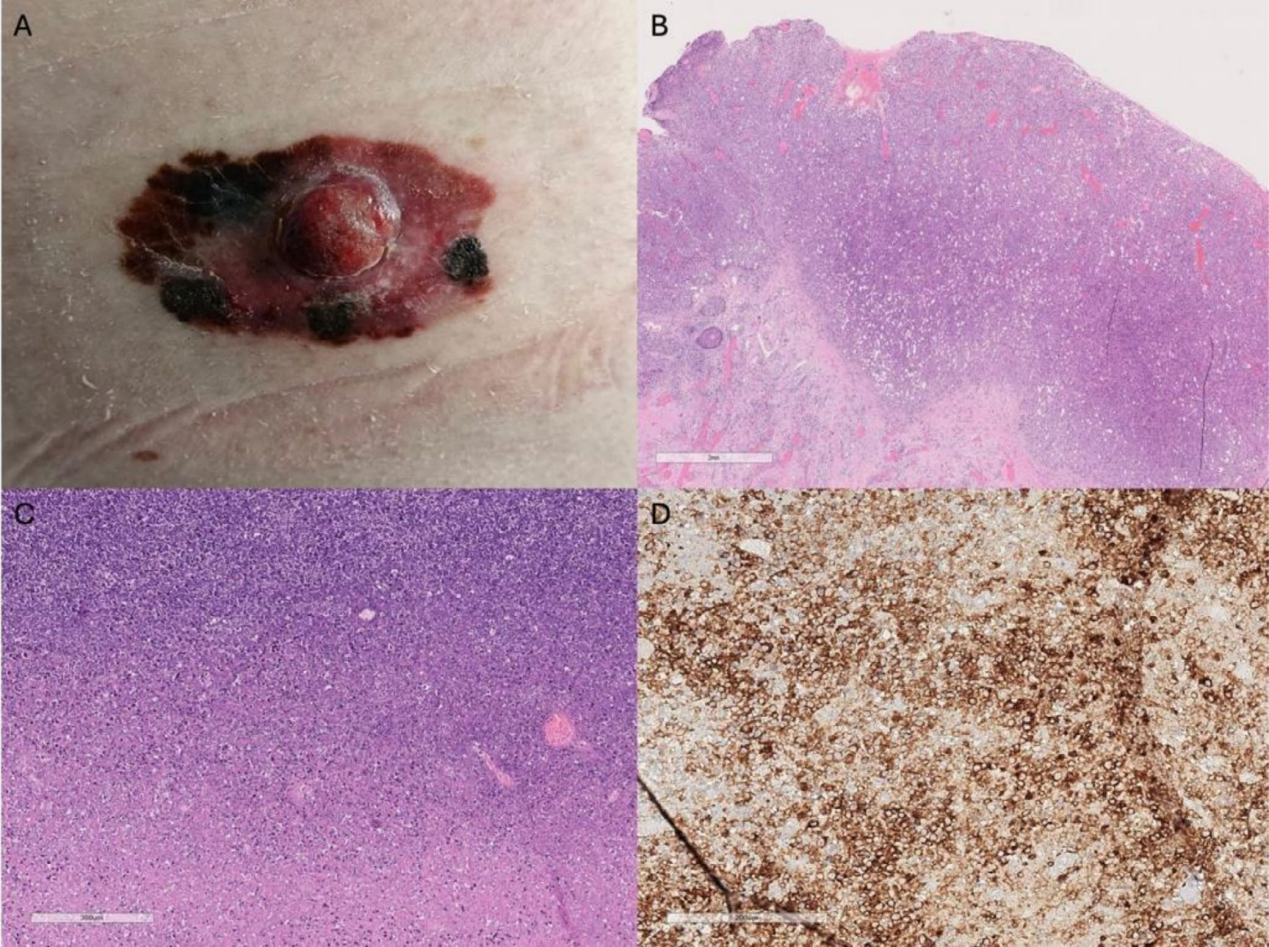
(A) Clinical presentation of SSM; (B) histopathology of SSM in vertical growth phase (H&E, 10×, scale bar represents 2 mm); (C) lymph node architecture is erased by a diffuse proliferation of neoplastic lymphoid infiltrate (H&E, 5× scale bar represents 300 μm); (D) CD20+ B‐cell aggregates within the involved lymph node (CD20, 10×).

**FIGURE 2:**
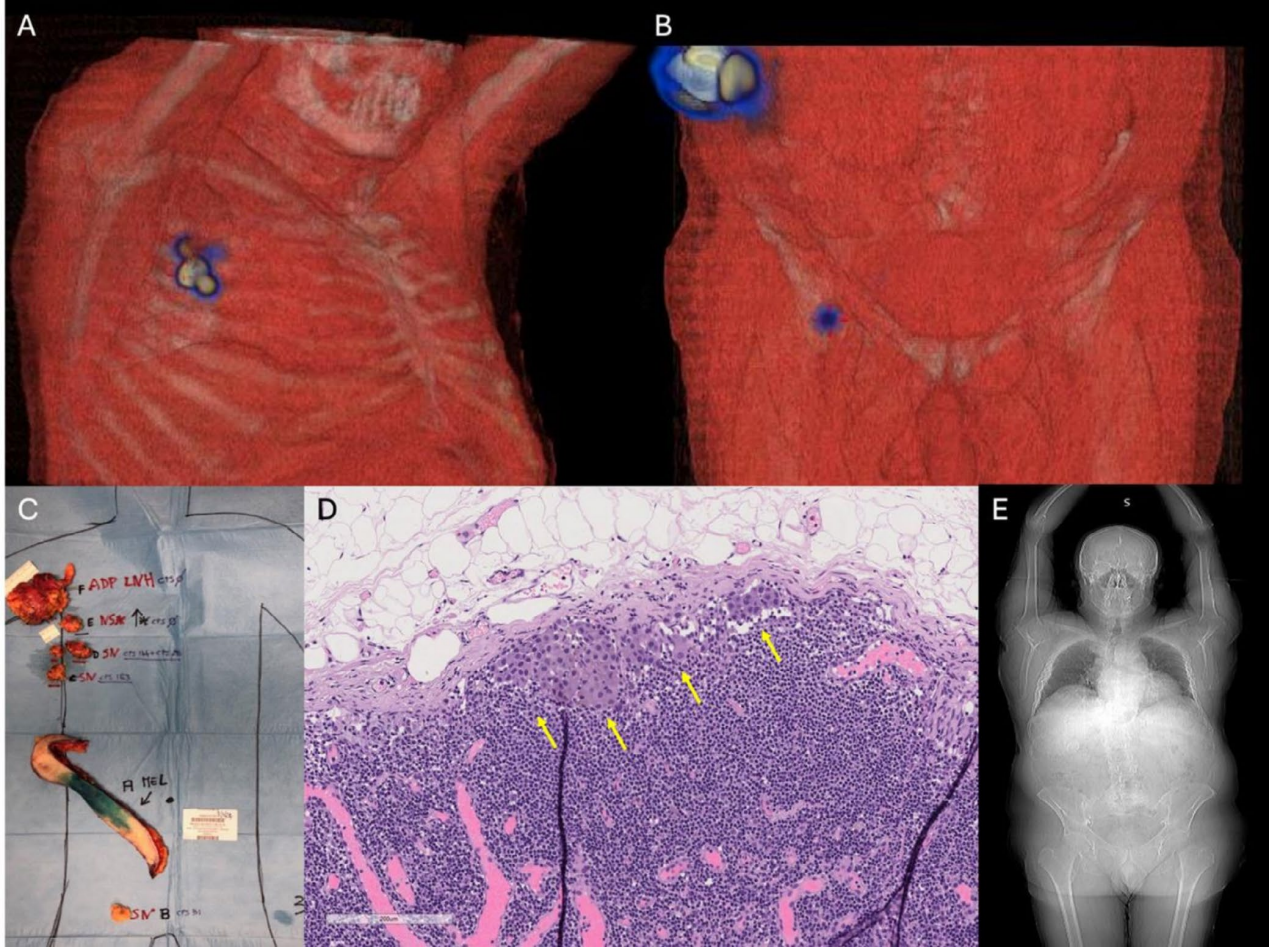
(A, B) SPECT/CT with 3D reconstruction showing radiotracer uptake in right axillary and inguinal nodes; (C) surgical specimens (a—wide excision of MM, b—right inguinal sentinel lymph node, c, d—right axillary sentinel lymph nodes; e—FL localization; f—FL previously biopsied adenopathies); (D) sentinel lymph node with melanoma metastasis (H&E, 10×, scale bar represents 200 μm); (E) contrast‐enhanced total‐body CT scan at 6‐month follow‐up showing no disease recurrence.

**FIGURE 3:**
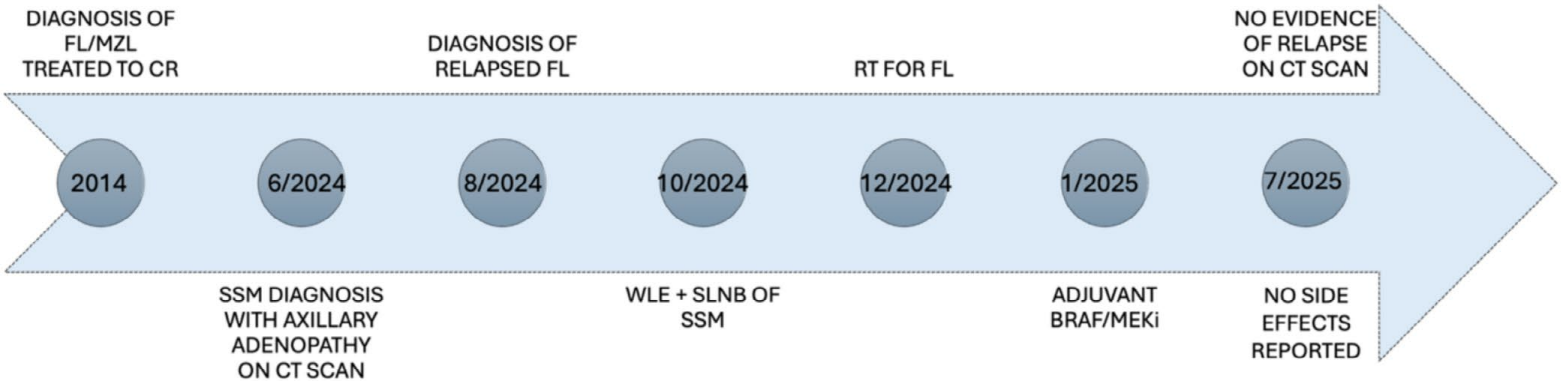
Clinical timeline of a 62‐year‐old male with a history of FL/MZL lymphoma treated with R‐CHOP, radiotherapy, and Rituximab maintenance, achieving long‐term remission. Ten years later, a high‐risk SSM was diagnosed. Staging revealed synchronous relapse of FL within the same axillary nodal basin. Management included WLE and SLNB for melanoma, RT for lymphoma, and finally adjuvant BRAF/MEK inhibitor therapy for melanoma. At 6‐month follow‐up, no evidence of recurrence was detected. CR, complete response; FL, follicular lymphoma; MZL, marginal zone lymphoma; RT, radiotherapy; SLNB, sentinel lymph node biopsy; SSM, superficial spreading melanoma; WLE, wide local excision.

A 62‐year‐old male with a medical history of hypertension, benign prostatic hyperplasia, and B‐cell lymphoma, exhibiting features intermediate between marginal zone and follicular subtype, with extradural lumbosacral localization, was treated with six cycles of R‐CHOP chemotherapy, followed by paravertebral radiotherapy (30 Gy) and a 2‐year maintenance with Rituximab, achieving a complete remission. During a 10‐year hematological follow‐up, no evidence of lymphoma recurrence was detected. Surveillance included serial PET‐CT‐FDG imaging during the initial 5 years, with subsequent monitoring limited to clinical evaluations. A decade later, in May 2024, the patient presented to the Dermatologic Clinic of the University of Turin, Italy, with a skin lesion clinically highly suggestive of MM, characterized by an irregularly pigmented patch‐to‐plaque with a central amelanotic nodule, measuring approximately 6 × 3 cm (Figure 1A). Surgical excision confirmed an ulcerated superficial spreading melanoma (SSM) exhibiting vertical growth. Histological examination revealed a Breslow thickness of 4.5 mm (pT4b, AJCC 8th edition), a mitotic rate of 35 mitoses/mm^2^, non‐brisk tumor‐infiltrating lymphocytes (TILs), and extensive regression (< 75%). Lymphovascular invasion was present, while perineural invasion was absent (Figure 1B). Staging contrast‐enhanced CT scan demonstrated a right axillary lymphadenopathy measuring 4 × 6 cm. No other distant locations were found. Core needle biopsy of the lymph node revealed lymphoreticular tissue with effaced architecture. The tissue was infiltrated by an atypical lymphoid cell population arranged in vague nodules. These nodules were composed of centrocyte‐like cells with scant cytoplasm, slightly irregular nuclei, and inconspicuous nucleoli, admixed with rare, larger centroblastic cells (not exceeding 5 per high‐power field). Immunohistochemical stains confirmed the B‐cell nature (CD20+) of the neoplastic cells, which co‐expressed CD10, BCL6, and BCL2, and were negative for CD43. Staining for CD21 highlighted a rarefied follicular dendritic cell meshwork. The MIB1 (Ki‐67) proliferation index was approximately 10%–15%. These findings were consistent with a diagnosis of FL. The patient then underwent wide local excision of the MM and sentinel lymph node biopsy (SNLB). Lymphoscintigraphy identified four radioactive nodes: one right inguinal and three right axillary (Figure 2A,B). During surgery, the previously biopsied node and an additional non‐sentinel right axillary node were removed (Figure 2C). Histopathological examination confirmed FL in the biopsied and non‐sentinel nodes, while melanoma metastasis was detected in one of the sentinel axillary nodes (Figure 2D). Bone marrow biopsy was negative for lymphomatous infiltration. The case was staged as IIIC melanoma (AJCC 8th edition) with concomitant relapsed FL confined to nodal sites. Molecular analysis of MM revealed a BRAFV600E mutation, and the patient was subsequently started on adjuvant targeted therapy with BRAF/MEK inhibitors, which was preferred over immunotherapy due to its more favorable long‐term tolerability profile [8]. Right axillary radiotherapy was delivered first due to the localized nature of the FL and was completed 20 days prior to the initiation of adjuvant therapy, to minimize the potential for increased skin toxicity [9, 10]. Six months after the initiation of adjuvant treatment, total body contrast‐enhanced CT scan showed no evidence of relapse of either MM or FL (Figure 2E). No adverse or unanticipated events were reported during the follow‐up period. The timeline of clinical events is illustrated in Figure 3.

## 3 Discussion

This case underscores the diagnostic and therapeutic complexity of synchronous malignancies involving both cutaneous and hematologic neoplasms within the same nodal region. Although rare, such occurrences highlight the need for a multidisciplinary approach and individualized treatment planning. The primary challenge was diagnostic. The initial finding of axillary lymphadenopathy in a patient with a new high‐risk melanoma could have been easily attributed solely to MM metastasis. However, the patient's medical history prompted a core needle biopsy, which unexpectedly revealed relapsed FL. This highlights the importance of obtaining a tissue diagnosis for suspicious lymphadenopathy, even when a likely cause is apparent. The therapeutic management was also challenging. Treatment had to address both a high‐risk, BRAF+ MM and a localized, relapsed indolent FL. The decision‐making process required a multidisciplinary team, including dermatologists, oncologists, hematologists, radiotherapists, and surgeons. The chosen strategy—localized radiotherapy for the lymphoma followed by systemic targeted therapy for the melanoma—was designed to maximize efficacy for both diseases while minimizing overlapping toxicities. Combining radiotherapy with BRAF/MEK inhibitors requires careful sequencing to reduce the risk of radiosensitization and severe skin toxicity [11]. While associations between MM and NHL are documented, cases of synchronous metastasis in the same lymph nodal basin are exceptionally rare. Moreover, most of the previously reported cases describing synchronous MM and NHL date back several years, when treatment options were limited, mainly restricted to interferon‐based therapies [12, 13]. Interferon‐alpha was used as adjuvant therapy for melanoma and, in rare cases, for lymphoid neoplasia, but this approach is now largely obsolete due to the advent of immune checkpoint inhibitors and targeted therapies for melanoma and novel agents for lymphoma [14, 15]. Management should be guided by prioritizing the malignancy with the greatest impact on survival—typically MM—while considering the interplay between immunosuppression and disease progression. Our case represents the first reported case where synchronous stage III BRAF‐mutated MM and FL were managed using targeted BRAF/MEK inhibitors for MM combined with localized radiotherapy for FL. This case highlights the diagnostic and therapeutic complexity of managing synchronous malignancies in the context of the actual therapeutic landscape of MM. The successful outcome in our patient, with no evidence of recurrence of either disease at 6 months of follow‐up, within the absence of side effects, validates the efficacy of a carefully planned, sequential, and multidisciplinary therapeutic approach. Limitations are the short follow‐up period, as both MM and FL can exhibit late relapses, and the generalizability of this sequential treatment approach that requires validation in larger case series and prospective studies. Given the reported association between lymphoid neoplasms and skin cancers, patients with a history of NHL should undergo long‐term dermatologic surveillance for early detection of cutaneous malignancies. In conclusion, this case illustrates a potential treatment approach for similar presentations while emphasizing the critical importance of individualized, multidisciplinary care in the management of synchronous malignancies, particularly within the evolving landscape of MM treatment.

## Author Contributions

Conceptualization: Silvia Borriello, Umberto Santaniello, and Franco Picciotto. Investigation: Giovanni Cavaliere, Adriana Lesca, Rebecca Senetta, and Matteo G. Brizio. Writing – original draft: Silvia Borriello and Umberto Santaniello. Writing – review and editing: Giulia Carpentieri and Paolo Fava. Supervision: Simone Ribero, Pietro Quaglino, and Franco Picciotto.

## Ethics Statement

The patient in this manuscript has given written informed consent to the publication of the case details including photographs.

## Conflicts of Interest

The authors declare no conflicts of interest.

## Data Availability

The data that support the findings of this study are available from the corresponding author upon reasonable request.
